# Supplementary material for: Mutations in respiratory complex I promote antibiotic persistence through alterations in intracellular acidity and protein synthesis
Source: Nat Commun. 2022 Jan 27;13:546. doi: 10.1038/s41467-022-28141-x (PMC8795404; doi:10.1038/s41467-022-28141-x)
Supplement: Supplementary file 1 — Supplementary Information [file 41467_2022_28141_MOESM1_ESM.pdf]

SUPPLEMENTARY INFORMATION

Mutations in respiratory complex I promote antibiotic persistence through alterations in intracellular acidity and protein synthesis

Bram Van den Bergh[1,2,3], Hannah Schramke[4], Joran Elie Michiels[1,2], Tom E.P. Kimkes[4], Jakub Leszek Radzikowski[4], Johannes Schimpf[5], Silke R. Vedelaar[4], Sabrina Burschel[5], Liselot Dewachter[1,2], Nikola Lončar[6], Alexander Schmidt[7], Tim Meijer[4], Maarten Fauvart[1,2,8], Thorsten Friedrich[5], Jan Michiels[1,2,9*], Matthias Heinemann[4,9*]

Supplementary Tables

Supplementary Table 1: Mutations found in randomly selected clones from evolved* E. coli *populations.

**Clone[1]**	**Genomic location[2]**	**Type of mutation**	**Reference**	**Allele**	**Gene**	**Amino acid change**
AMK-1-1[3]	1303437	SNV	C	A	*oppB*	Ala180Glu
AMK-1-2	1303437	SNV	C	A	*oppB*	Ala180Glu
AMK-1-3	1303437	SNV	C	A	*oppB*	Ala180Glu
AMK-1-4	1303437	SNV	C	A	*oppB*	Ala180Glu
AMK-1-5	1569851	Insertion	-	AAT	*gadC*	Ser213SerTyr
AMK-2-1[3]	2390302	SNV	C	G	*nuoN*	Gly402Arg
AMK-2-2	3438087	SNV	G	A	*mscL*	Gly22Ser
AMK-2-3	1303437	SNV	C	A	*oppB*	Ala180Glu
AMK-2-4	1303437	SNV	C	A	*oppB*	Ala180Glu
AMK-2-5	1303437	SNV	C	A	*oppB*	Ala180Glu
AMK-3-1	1303437	SNV	C	A	*oppB*	Ala180Glu
AMK-4-1	823950	SNV	G	A	*ybhP*	Ala183Asp
AMK-4-1	1303437	SNV	C	A	*oppB*	Ala180Glu
AMK-5-1	3438102	Insertion	-	GGT	*mscL*	Ala27GlyAla
AMK-6-1[3]	1569390	SNV	A	G	*gadC*	Phe367Ser
AMK-7-1	2390616	SNV	A	C	*nuoN*	Leu297Arg
AMK-8-1	45934	SNV	A	C	*yaaU*	Gln43Pro
AMK-8-1	3149003	SNV	C	A	*yqhA*	Asp157Tyr
AMK-9-1	1569858-1569872	Deletion	ATGAAGGCAACAAAT	-	*gadC*	Phe207_Ile211del
AMK-10-1	722427	SNV	A	C	*kdpD*	Ile663Ser
AMK-10-1	1303437	SNV	C	A	*oppB*	Ala180Glu
KAN-1-1	2394166	SNV	G	T	*nuoL*	Ala294Asp
TOB-1-1[4]	1569375-1569380	Deletion	GCCAGT	-	*gadC*	Ala372_Leu373del
GEN-1-1[4]	2390577	SNV	A	C	*nuoN*	Leu310Arg
UTI-1-1	2524117	SNV	A	C	*nuoM*	Phe356Cys
UTI-1-2	2524117	SNV	A	C	*nuoM*	Phe356Cys
UTI-2-1	2523412	SNV	A	C	*nuoN*	Leu79Arg
UTI-3-1	2523942	SNV	G	T	*nuoM*	Ser414Arg
UTI-3-1	3058278	SNV	T	G	*YP542126*	Asn65Glu

In total, 23 clones were assessed by whole-genome sequencing. In total we found 29 mutations in 11 different genes. The limited number of mutations per clone (1 or 2) agrees with the limited evolutionary time (<100 generations) and the good fit of a model for the spread of one mutant in a wild-type background to the evolutionary changes (see Supplementary Fig. 1a, and Fig. 1 in Van den Bergh *et al.*, 2016[1]). 19 mutations are unique with a single mutation in *oppB* reoccurring in multiple clones. Respectively 4 and 3 clones from the first and second amikacin-evolved population harbor the same mutation in oppB (Supplementary Data 1). The same mutation is found in other independent amikacin evolved clones and in 12 populations in total (Supplementary Fig. 1b). Additionally, *mscL* and *gadC* also qualify as strong evolutionary targets as respectively 2 and 4 different alleles of the genes were found, each in different populations. Most interesting are mutations that accumulated in nuo. In total, 8 clones contained a mutation in *nuoLM* or *N*, both in the lab strain and in the UTI89 background and throughout

samples from 3 out of 4 aminoglycosides. It is important to note that none of these alleged targets were found to be mutated together in the same clonal background.

[1]Naming is as follows: the first abbreviation denotes the antibiotic that was used during the evolution experiments, the first number indicates the population of origin and the last number is the clone identifier.

[2]Location with regards to the used references, either *E. coli* K12 MG1655 NC_000913.3 or *E. coli* UTI89 NC_007946.1 (genome) and NC_007941.1 (plasmid).

[3]These samples were analyzed and published before by Van den Bergh et al., 2016[1] but are included here for comparison.

[4]While genotype information of most clones was obtained by whole-genome-sequencing using Illumina Technology, data for these clones come from targeted resequencing through Sanger's technology.

***Supplementary Table 2: Mutations found in the* nuo *operon across all evolved* E. coli *populations*.**

Gene	Residue[1]	AA reference[1]	AA variant	# populations	cum freq	Hydrophilic change[2]?	Transmembrane section[3]?
nuoA	73	Met	Lys	2	3.53	1	1
nuoA	78	Phe	Ser	2	2.66	1	1
nuoA	85	Leu	Arg	1	15.56	1	1
nuoB	70	Thr	Ala	1	1.01	0	0
nuoB	82	Gly	Ser	1	1.26	1	0
nuoC	200	Trp	Gly	1	1.35	1	0
nuoG	404	Met	Arg	1	1.19	1	0
nuoG	567	Leu	Val	1	1.56	1	0
nuoG	588	Pro	fs	1	1.41	-	0
nuoH	40	Leu	Arg	1	10.74	1	0
nuoJ	54	Leu	Arg	2	30.22	1	1
nuoJ	149	Leu	Arg	1	1.09	1	1
nuoK	28	Leu	Arg	3	16.34	1	1
nuoK	67	Ser	Arg	1	45.26	1	1
nuoK	77	Leu	fs	1	1.10	-	1
nuoL	98	Leu	His	1	1.26	1	1
nuoL	171	Phe	fs	1	2.52	-	1
nuoL	239	Leu	Arg	1	1.13	1	0
nuoL	290	Thr	Met	1	1.23	0	1
nuoL	294	Ala	Asp	1	2.62	1	1
nuoL	417	Ala	Ser	1	1.33	1	1
nuoL	418	Gly	Val	1	1.65	0	1
nuoL	421	Gly	Asp	1	1.45	1	1
nuoL	423	Phe	Ser	1	1.53	1	1
nuoM	190	Leu	Arg	1	5.97	1	1
nuoM	247	Ala	Glu	1	2.43	1	0
nuoM	316	Ala	Asp	1	1.66	1	1
nuoM	337	Ala	Val	1	1.37	0	0
nuoM	342	Val	Glu	5	129.78	1	0
nuoM	356	Phe	Cys	6	205.36	1	1
nuoM	356	Phe	Ser	2	6.15	1	1
nuoM	414	Ser	Arg	1	11.72	1	0
nuoN	26	Leu	Arg	1	1.31	1	1
nuoN	79	Leu	Arg	2	81.15	1	1
nuoN	128	Leu	Arg	1	6.58	1	1
nuoN	229	Pro	del	1	98.66	-	0
nuoN	230	Val	Glu	1	3.11	1	0
nuoN	234	Ala	Glu	3	105.02	1	1
nuoN	297	Leu	Arg	14	287.79	1	1
nuoN	310	Leu	Arg	2	67.32	1	1
nuoN	330	Leu	Arg	1	1.46	1	1
nuoN	402	Gly	Asp	6	110.56	1	0

nuoN	402	Gly	Arg	1	3.70	1	1
nuoN	424	Tyr	Asp	2	46.85	1	1
nuoN	474	Leu	Arg	6	129.54	1	1
nuoN	340-342	AlaPheGly	del	4	46.61	-	1
nuoN	341-343	PheGlyVal	del	1	1.33	-	1

Gray entries are mutations that only were detected below 5% frequency in their populations.

[1]Mutations from the UTI background were added based on a pairwise alignment to the lab strain background.

[2]1=yes, 0=no. Deduced from comparing hydrophobicity values as calculated as done before[3]

[3]1=yes, 0=no. Deduced from Protter output[4]

Supplementary Figures

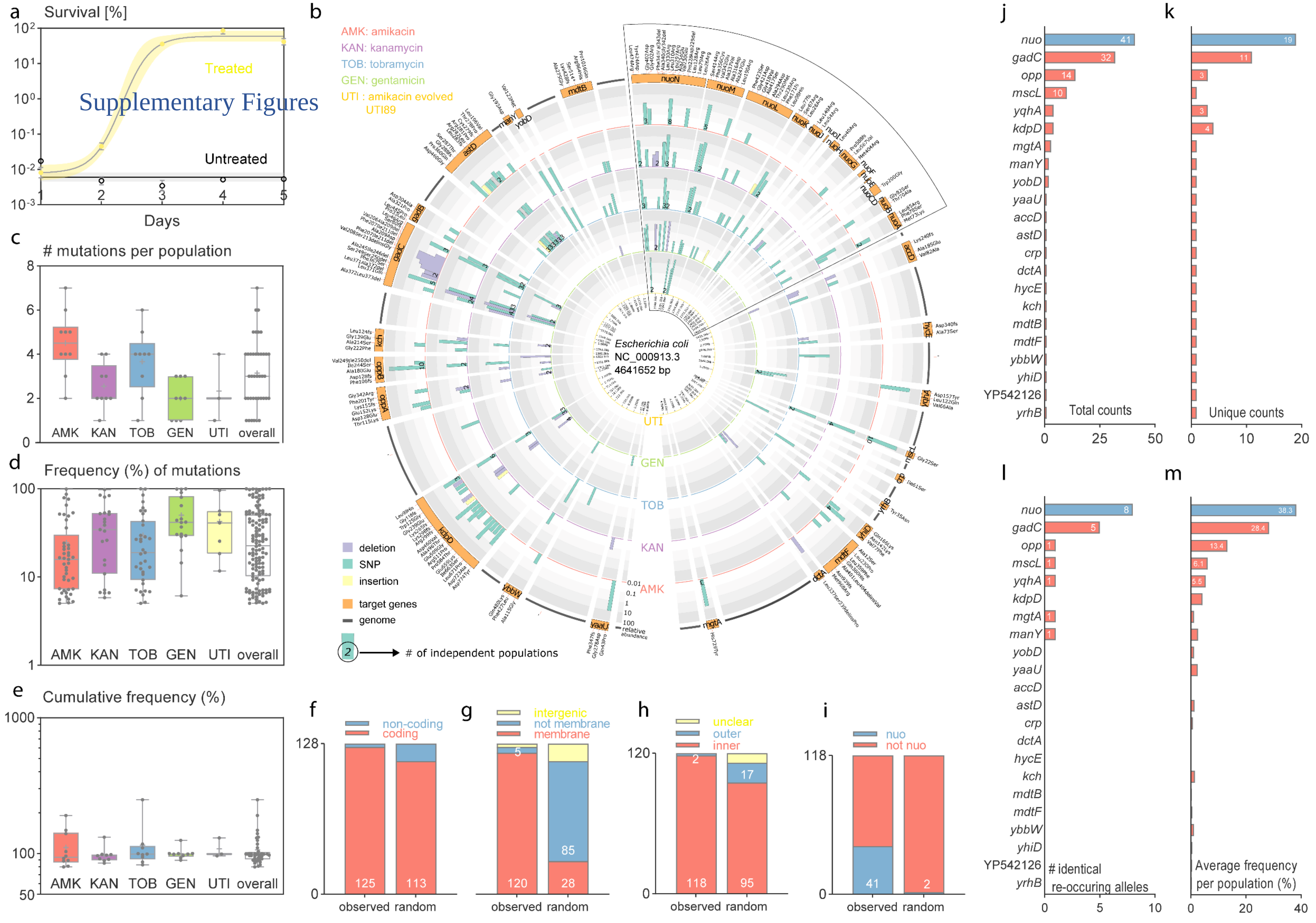

Supplementary Figure 1 – *E. coli* populations that evolve under daily antibiotic treatment increase in persistence by acquiring mutations in the *nuo* operon, Related to Fig. 1. a The number of persisters in the uropathogenic strain UTI89 increases during evolutionary adaptation under daily amikacin treatments (for 5h at 400 µg ml^{-1}) as in Fig. 1a (yellow; a model of the spread of a single mutation was fitted to the data with 95% shaded confidence interval; means ±sems, n=3). Evolution without antibiotic treatment (black) has no effect on the persistence level as a horizontal line with a slope = 0 fits better to the data than a straight line with slope ≠ 0 (F test; with 95% shaded confidence interval). For details on fittings, see Methods. **b** A circos plot visualizes mutations that emerged in all populations evolved under Fig. 1a. The concentric circles show the average frequency of mutations in populations evolved on different antibiotics and in populations of the uropathogenic strain UTI89 evolved in **a**. Formatting is as in Fig. 1b. The insert highlights the *nuo* operon which is enlarged in Fig. 1b. **c-e** Statistics of **c** number of mutations, **d** frequency of mutations and **e** cumulative frequency in each population per used antibiotic, for the UTI89 populations and overall. Data of ten (amikacin), nine (kanamycin, tobramycin, and gentamicin) and three (UTI89) evolved populations were cumulated with the number of mutations as in Supplementary Data 1. Boxes extend from the 25th to 75th percentile with a line at median and with whiskers showing min and max. Cumulative frequencies lie around 100% which further indicates that the wild type has almost been fully replaced by mutants, also given the fact clones seldomly carry more than 1 mutation and clones without mutations were not found (see Supplementary Table 1). Populations with a cumulative frequency above 100% likely contain double mutants while cumulative frequencies below 100% indicate undetected minor alleles or a remaining low percentage of wild types. Boxes show 25-75th percentiles (edges), median (line) and min and max (whiskers). **f-i** Statistically significant enrichment (p <0.0 of mutations in **f** coding regions, genes coding for **g** membrane proteins and specifically for **h** inner membrane proteins and in **i** the *nuo* operon (for f-i, $p < 0.05$ based on Chi^2 comparisons to random mutations, see Methods). Numbers in the bars show the number of events for the largest (and second largest) groups. The random null hypothesis was generated by assuming random chance of mutation across the genome with corrections for the sizes of the respective groups. **j-m** Statistics per target gene (in red) across populations to show that *nuo* (in blue) is the main evolutionary target with **j** the total mutation events, **k** the number of unique mutations,

l the number of different, re-occurring identical alleles and **m** the average cumulative frequency of all mutations per target per population. **b** and **m** were generated using both the mutations above 5% ànd those in identified targets below 5% of frequency while **c-l** use only mutations with a frequency above 5%. Source data are provided as a Source Data file.

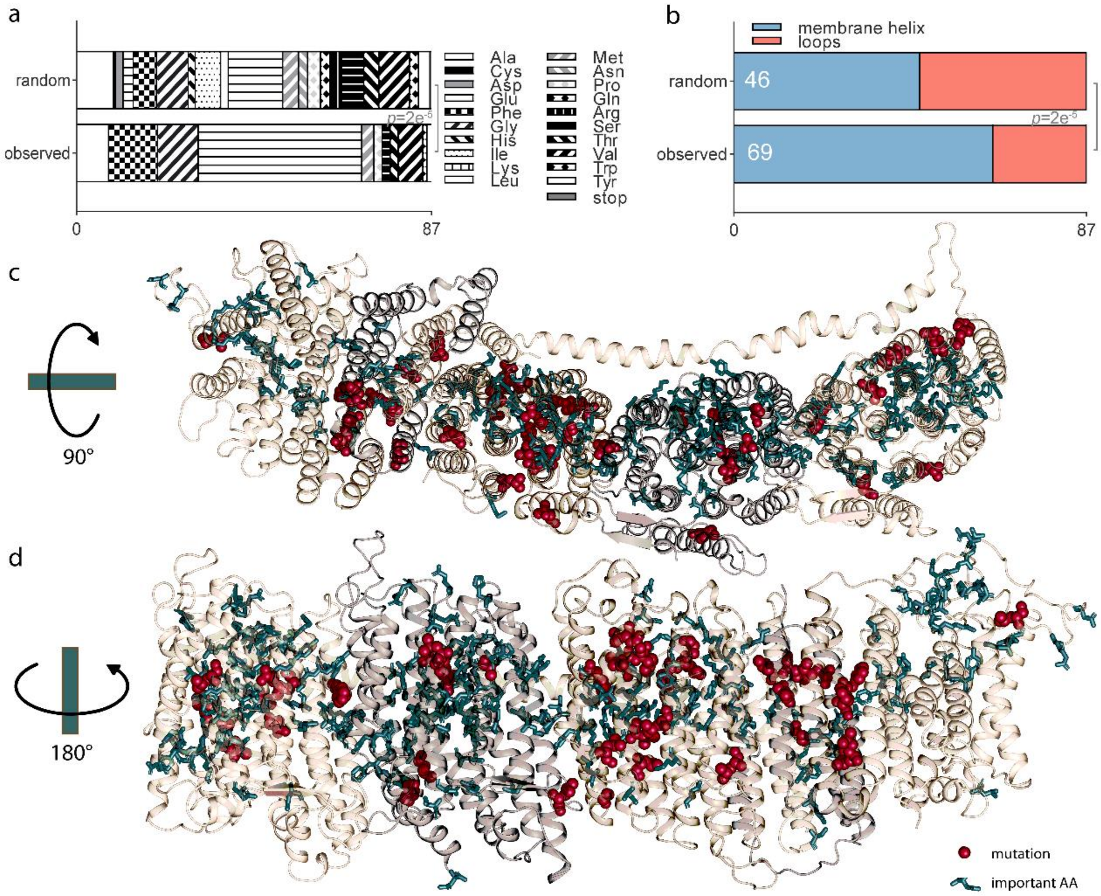

e

	Amikacin (µg/ml)	Kanamycin (µg/ml)	Ofloxacin (µg/ml)
WT	2-4	8	0.064
*L** A294D	2	8	0.064-0.128
*M** F356C	2-4	8	0.064
*N** G402R	4	4-8	0.064
ΔL	2-4	4	0.032
ΔM	2-4	4	0.032
ΔN	2-4	4-8	0.032-0.064
Δnuo	2-4	4-8	0.032

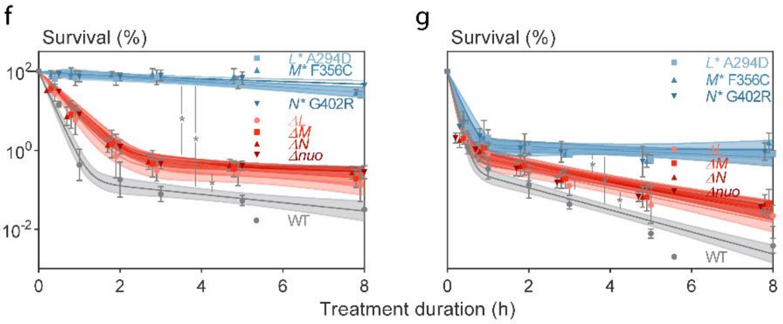

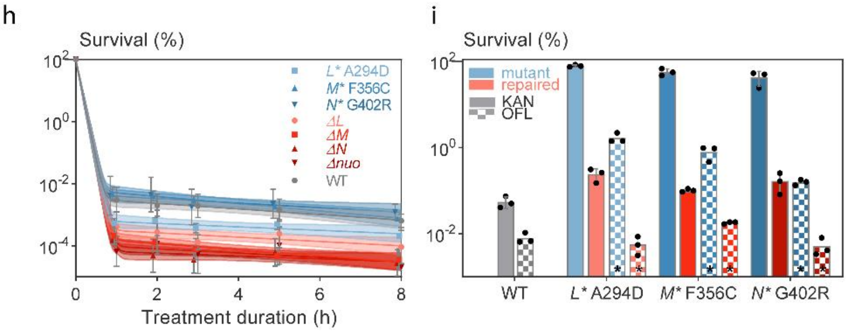

Supplementary Figure 2 – Mutations in genes encoding membrane subunits of *nuo* are highly specific and cause multidrug tolerance without increased resistance, Related to Fig. 2. a and **b** The identified mutations in *nuoAHJKLMN* hit specific amino acids **a** which are predominantly part of membrane-spanning helices **b** (from Chi² comparisons to random mutations, see Methods). **c** and **d**

Different orientations of the magnified membrane part of the inset in Fig. 2a, with a view from the bottom (**c**; 90° upward rotation) and behind (**d**; 180° rightward rotation) with high persister variants (red spheres) and important residues for the functioning of the H^+-pathways (blue sticks) (from Baradaran et al., 2013; Efremov and Sazanov, 2011; Di Luca et al., 2017[19–21]). **e** Minimum inhibitory concentrations for all mutants and antibiotics show that increased resistance does not explain the increased survival in these mutants. **f-h** Killing dynamics show cross-tolerance of the mutants towards **f** kanamycin (400 µg ml^{-1}) and **g** ofloxacin (5 µg ml^{-1}) in stationary phase while knockout mutants in a single gene or the entire operon (in red) only show marginal increase in persistence and **h** tolerance towards ofloxacin in exponential phase (5 µg ml^{-1}) is not increased. A model describing biphasic killing dynamics (95% shaded confidence interval) was fitted to the data (means ±stdevs, n=3; * fits of groups are different based on AIC criterion). **i** As for amikacin tolerance in Fig. 2e, tolerance to kanamycin (5h at 400 µg ml^{-1}; full bars) and ofloxacin (5h at 5 µg ml^{-1}; hatched bars) is lost when mutations in subunits L, M and N are genomically repaired (red bars) (mean ±stdevs, n=3; * in the bars: p<0.0001 three-way ANOVA with Tukey's test to the unrepaired strain). Source data are provided as a Source Data file.

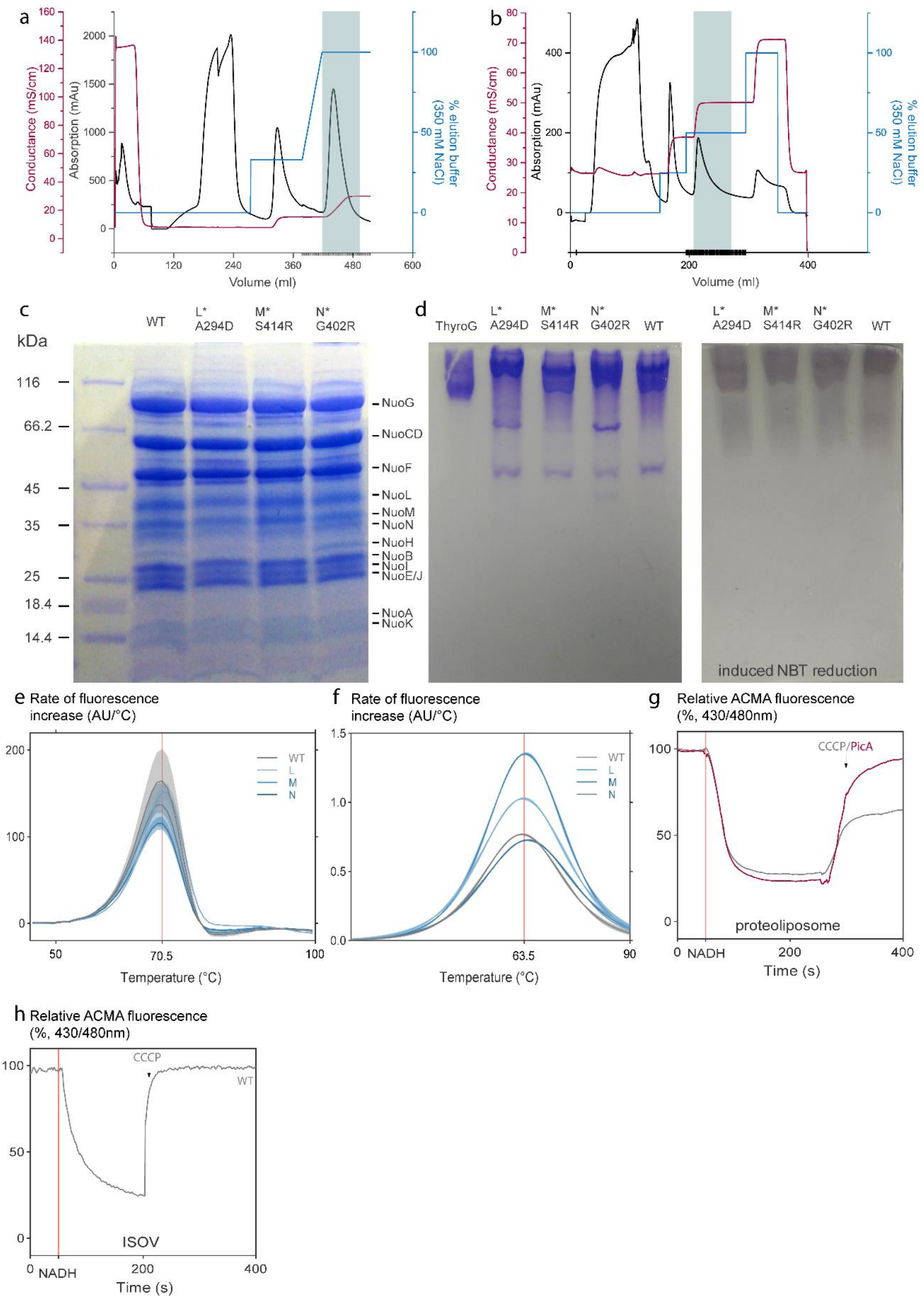

Supplementary Figure 3 – High persistence-conferring variants of NuoL, M and N were purified as intact, stable complex I with similar NADH oxidase activity as the wild type, Related to Fig. 3. **a** and **b** The main chromatographic steps used in the purification of complex I and its variants are **a**

anion exchange chromatography on a Fractogel EMD TMAE Hicap (Merck) and **b** affinity chromatography for the His_6-tag labelled complex I on Ni-IDA material (Invitrogen) (see Methods). Representative chromatographs are shown. Boxed regions indicate NADH/ferricyanide oxidoreductase active fractions that were pooled and taken along in the purification. Small ticks on the x axis show the fractions that were sampled. **c** and **e** Gel separation of purified complex I using a **c** denaturing SDS PAGE (75 µg) and **d** Clear-native PAGE (CN-PAGE) (40 µg). **c** After Coomassie staining, variants and wild type show similar band patterns of subunits (bands were assigned to individual units based on size comparison on the right). Left lane contains Pierce unstained protein marker (Thermo Scientific). **d** The majority of the purified complexes remains stable upon purification (left, highly dense Coomassie-blue bands on the top; Thyroglobulin is added in the first lane as size comparison on this native, non-denaturing, gel) and is capable of *in situ* reduction of the dye NBT by NADH oxidation (right gel, strong black signal on the top) (gels in **c** and **d** are run once). **e** and **f** Stability of the purified complexes was further confirmed by "melting point" determination based on **e** the release of autofluorescent flavin mononucleotide, the catalytic co-factor of complex I, and **f** the binding of CPM, which increases in fluorescence upon reaction with thiol groups (of cysteines) that are released upon heating and unfolding. In **e** and **f** the first-order derivatives are shown (means ±sems error bands, n=3). "Melting points" are identified as peaks and shown by a vertical red line and number. **g** and **h** The ACMA quench in proteoliposomes or ISOVs (Fig. 3c, d) is a measure of the proton gradient generated by complex I. In proteoliposomes of wild-type complex I **g** the fluorescence quench is reverted by adding either the uncoupler CCCP (gray) or the specific complex I inhibitor piericidin A (red). CCCP does not fully revert the quench as CCCP itself contributes to the measured fluorescence is this assay. The experimental conditions were the same as in Fig. 3c. **h** For ISOVs containing wild-type complex I, the ACMA quench of the IMV is fully reverted by the uncoupler CCCP. With the substantially higher protein concentration in the assay than in **g**, the fluorescence of CCCP is not detectable. The experimental conditions were the same as in Fig. 3d. Source data are provided as a Source Data file.

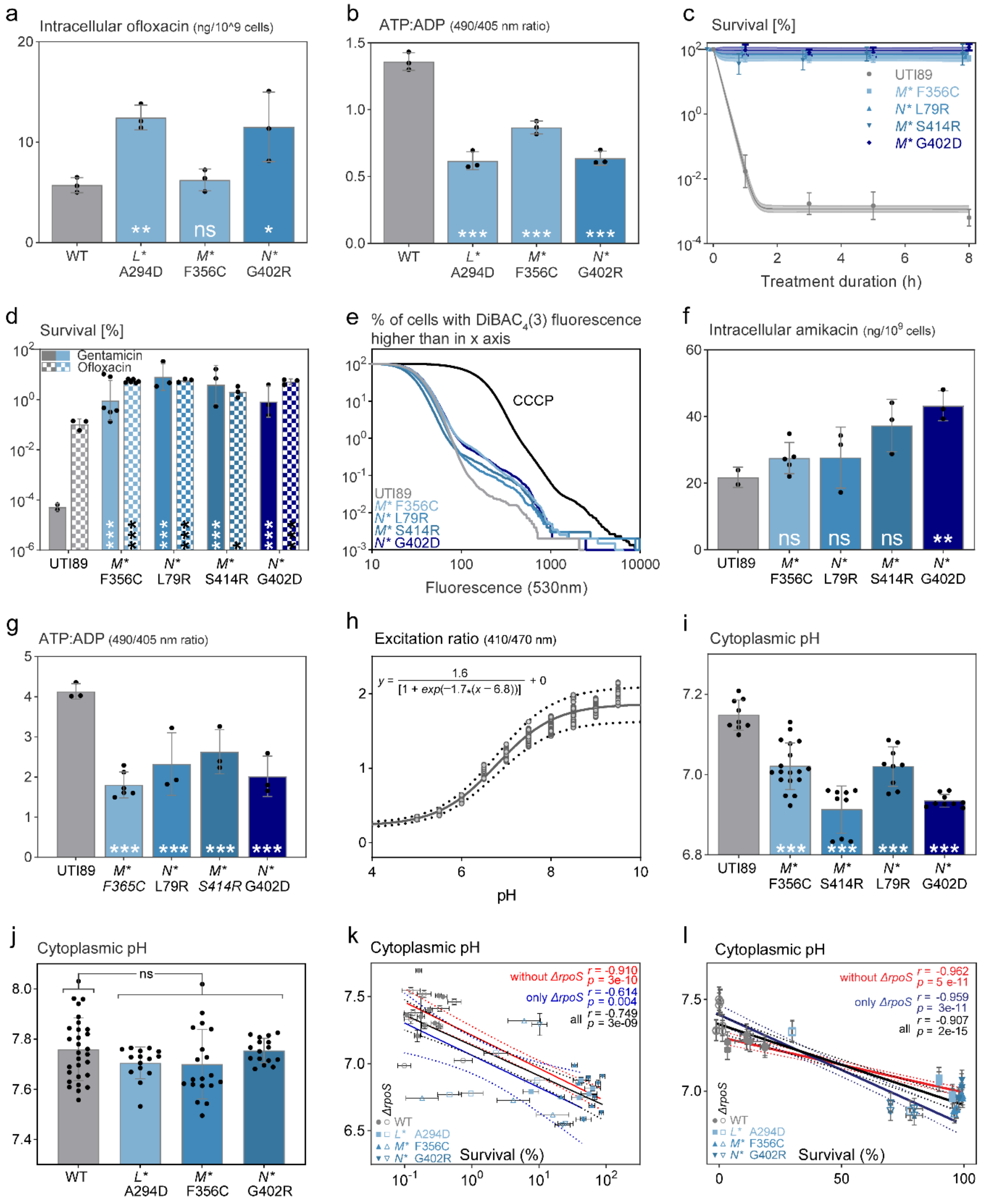

Supplementary Figure 4 – Increased persistence in the identified *nuo* mutants does not depend on impaired antibiotic uptake or a decreased energy status, Related to Fig. 4-6. a Uptake of ofloxacin is not decreased in the *nuo* mutants. Furthermore, a decreased uptake through decreased PMF would not explain the multidrug tolerance to this class of antibiotics as the uptake of fluoroquinolones is not powered by PMF (means ±stdevs, n=3; ** p<0.01 and * p<0.05 of a one-way ANOVA model

with Dunnett's multiple comparisons test to the wild type). **b** The ATP:ADP ratio in exponential phase is significantly decreased in all mutants in *nuo* as measured in populations by fluorescent ratiometry after expressing the ATP-sensitive fluorophore Perceval (means ±stdevs, n=3; **** p<0.0001 of a one-way ANOVA model with Dunnett's multiple comparisons test to the wild type). **c-g** and **i** Measurements on *nuo* mutants in the UTI89 background showing **c** increased persistence for amikacin (400 µg ml^{-1}) during a time-kill curve (a biphasic killing model ±95% shaded confidence interval was fitted to the data; n=3), **d** cross tolerance to a 5 hour treatment with gentamicin (400 µg ml^{-1}) and ofloxacin (5 µg ml^{-1}) (n≥3), **e** minor changes in electric potential as measured by the uptake of $DiBAC_4(3)$, a potential-sensitive fluorescent dye, assessed on the single-cell level using flow cytometry (the 100% - cumulative distribution is plotted in function of fluorescence showing which fraction of the population has a fluorescence higher than the x value), **f** an unchanged amikacin uptake as measured by a bioassay, **g** a significant decrease of the ATP:ADP ratio in stationary phase assessed with Perceval and **i** a significant internal acidification at stationary phase measured using pHluorin. For **c-d, f-g** and **i**, means ±stdevs, n=3 and for **e** one representative repeat of distributions are shown for n=2. For **d**, * p<0.05, and *** p<0.0001 from a two-way ANOVA model with Dunnett's multiple comparisons test to the wild type. For **f, g, i**, ** p<0.01, *** p<0.001 from a one-way ANOVA model with Dunnett's multiple comparisons test to the wild type. **h** Example of calibration curve for pH calculations. *E. coli* cells expressing pHluorin were washed and resuspended in M63 minimal salts medium at different pH values containing 40 mM benzoate and 40 mM methylamine. Next, the Boltzmann equation (line ±95% PI, n=48) was fitted to the excitation ratio (410/470nm) data and used as calibration curve. **j** *nuo** mutants in the lab strain in exponential phase with an external pH of 7.12 do not show general internal acidification as they do in stationary phase (Fig. 5a; means ±stdevs, n=3; ns = non-significant for a phenotype-level comparison from a mixed-effects model). **k** and **l** Cytoplasmic pH shows significant negative correlation with survival of amikacin treatment (400 µg ml^{-1}) both in **k** stationary phase and **l** 30 min after the shift from glucose to fumarate, regardless of whether all strains (black) or only strains with/without functional *rpoS* (red/blue; closed/open symbols) are considered (linear regressions ±95% CIs, Pearson r and p values in top right corner; means ±sems, n≥4). **l** Shows the data of strains with

functional *rpoS* from Fig. 5c for visual comparison and correlates survival to the cytoplasmic pH at 220 min after the switch (means ±sems, n≥6). Source data are provided as a Source Data file.

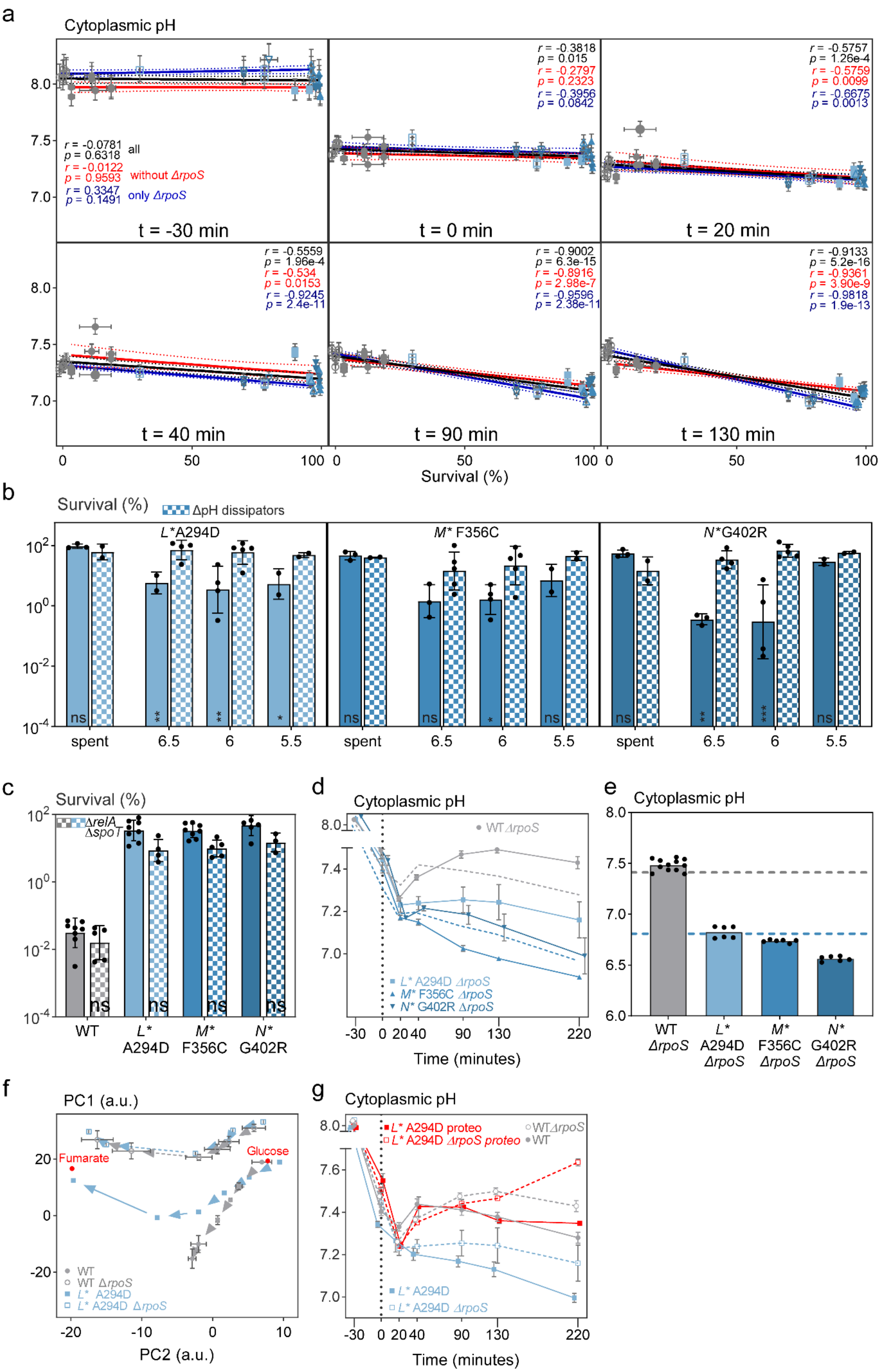

a
Cytoplasmic pH
r = -0.0781
p = 0.6318
all
r = -0.0122
p = 0.9593
without ΔrpoS
r = 0.3347
p = 0.1491
only ΔrpoS
t = -30 min
r = -0.3818
p = 0.015
r = -0.2797
p = 0.2323
r = -0.3956
p = 0.0842
t = 0 min
r = -0.5757
p = 1.26e-4
r = -0.5759
p = 0.0099
r = -0.6675
p = 0.0013
t = 20 min
r = -0.5559
p = 1.96e-4
r = -0.534
p = 0.0153
r = -0.9245
p = 2.4e-11
t = 40 min
r = -0.9002
p = 6.3e-15
r = -0.8916
p = 2.98e-7
r = -0.9596
p = 2.38e-11
t = 90 min
r = -0.9133
p = 5.2e-16
r = -0.9361
p = 3.90e-9
r = -0.9818
p = 1.9e-13
t = 130 min
Survival (%)
b
Survival (%)
ΔpH dissipators
L*A294D
M* F356C
N*G402R
spent
6.5
6
5.5
ns
c
Survival (%)
ΔrelA ΔspoT
WT
L* A294D
M* F356C
N* G402R
d
Cytoplasmic pH
WT ΔrpoS
L* A294D ΔrpoS
M* F356C ΔrpoS
N* G402R ΔrpoS
Time (minutes)
e
Cytoplasmic pH
WT ΔrpoS
L* A294D ΔrpoS
M* F356C ΔrpoS
N* G402R ΔrpoS
f
PC1 (a.u.)
Fumarate
Glucose
WT
WT ΔrpoS
L* A294D
L* A294D ΔrpoS
PC2 (a.u.)
g
Cytoplasmic pH
L* A294D proteo
L* A294D ΔrpoS proteo
WT ΔrpoS
WT
L* A294D
L* A294D ΔrpoS
Time (minutes)

Supplementary Figure 5 – Intracellular acidification underlies increased persistence in *nuo* mutants, Related to Fig. 4-6. a Survival of amikacin treatment (4h at 400 µg ml^{-1}) 30 min after the shift from glucose to fumarate negatively correlates significantly with cytoplasmic pH, starting from 20-40 min after the switch with correlations becoming stronger at later time points. Before (at -30 min or 0 min), correlations are non-significant or weak. The correlations are present regardless of whether all strains (black) or only strains with/without functional *rpoS* (red/blue; closed/open symbols) are considered (linear regressions ±95% CIs, Pearson r and p values in corners; means ±sems, $n \geq 6$). **b** As in the wild type (Fig. 5d), cytoplasmic acidification (boxed bars with ΔpH dissipators benzoate and methylamine at 40 mM) increased the survival of amikacin treatment (5h at 400 µg ml^{-1}) in each of the *nuo** mutants. Given the already high persister level of the *nuo** mutants, the effect of cytoplasmic acidification was much more modest than in the wild type. Adding this weak acid-base pair does not influence tolerance levels in unbuffered spent medium (means ±stdevs, $n \geq 2$; * $p<0.05$, ** $p<0.01$, *** $p<0.001$, and ns = non-significant for within-strain comparisons between presence/absence of dissipator for each pHe from a mixed-effects model with Šídák's posttest). **c** The increased persistence of the *nuo** mutants (5h amikacin treatment at 400 µg ml^{-1}) is independent of the alarmone (p)ppGpp as a knockout of *relA* and *spoT* has in non-significant effect (means ±stdevs, $n \geq 3$; ns = non-significant for within-strain comparison from a mixed-effects model). **d** and **e** The cytoplasmic pH of strains lacking *rpoS* is similar to pH of strains with *rpoS* **d** during a shift from glucose to fumarate and **e** in stationary phase. The average cytoplasmic pH of the wild type (gray) and all *nuo** mutants together (blue) are added as dotted lines as comparison in both plots (for **d,** means ±stdevs, $n \geq 4$; for **e,** means, $n \geq 6$). **f** and **g** A deviating experimental run with the *nuoL** mutants further substantiates our findings on how cytoplasmic acidification leads to antibiotic tolerance by halting proteomic changes. In this particular, single run, the *nuoL** mutants with and without *rpoS* (in respectively full/empty blue symbols and full/dashed lines) shows **f** a shift in proteome that is normal for steady-state growth on fumarate, likely because **g** acidification was not strong and persistent the proteome is not blocked and shifts towards a proteome. In **f** the wild type is added as comparison (with means ±stdevs, $n=3$). In **g** the deviating experimental run of the *nuoL** mutants is shown in red and compared to the average *nuoL** mutans in blue and wild type in gray (means ±sems, $n \geq 4$). Source data are provided as a Source Data file.

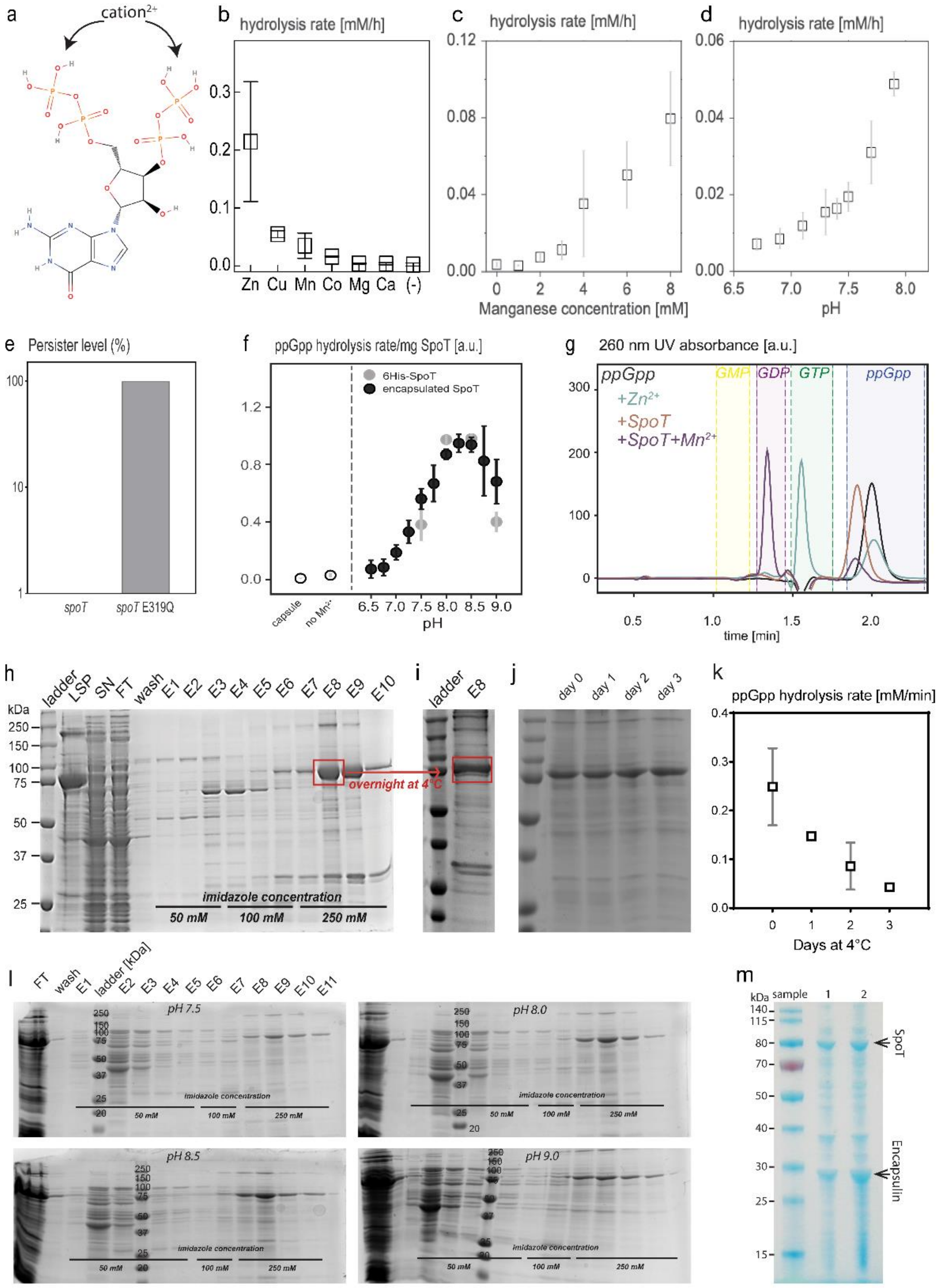

Supplementary Figure 6 - Rate of spontaneous and SpoT-dependent ppGpp hydrolysis depends on manganese and pH, Related to Fig. 6. a 2D structure of ppGpp (PubChem) and the potential divalent cation coordination sites are indicated with arrows. **b-d** Rate of spontaneous hydrolysis (*i.e.* slope of a linear regression fitted to the time-dependent ppGpp concentration data) is dependent on **b** type of divalent cation, **c** on the concentration of manganese and **d** on pH. For **b** and **c,** pH = 7.7, with

in **b** concentrations of divalent cations at 1 mM. For **d**, 4 mM manganese was used. All reactions were performed at 37°C with an initial ppGpp concentration of 1 mM. At higher pH values than 7.9 we observed precipitation of Mn^{2+}, therefore these data were discarded (means ±stdevs, n≥3). **e** The persister level of a strain with wild-type *spoT* does not differ from a strain carrying *spot* E319Q, a SpoT variant defective in ppGpp synthesis (means ±stdevs, n=3; p= 0.2005, t-test). **f** SpoT-dependent ppGpp hydrolysis rates determined by the slope of a linear regression fitted to the time-dependent ppGpp concentration data in buffers with different pH values. For samples with encapsulated SpoT (purified in **l**), rates were normalized to SpoT content in the sample. The reactions with the empty capsule and without Mn^{2+} were performed in reaction buffer at pH 8.0. All reactions were performed at 25°C in the presence of 1 mM Mn^{2+} with an initial ppGpp concentration of 1 mM (means ±stdevs, n≥3). For data of regularly purified SpoT, we eluted the protein from the Ni-Sepharose directly with buffers adjusted to the corresponding pH values due to low SpoT concentration and the significant loss of protein during buffer exchange. Here, only buffers between pH 7.5 and 9.0 could be used as the elution efficiency is pH dependent and 6His-SpoT only eluted at these pH values (**l**). **g** SpoT-dependent ppGpp hydrolysis critically needs manganese and results in GDP whereas spontaneous hydrolysis using metal ions results in GTP. HPLC-UV chromatograms with GMP, GDP, GTP and ppGpp standards (shades), ppGpp in buffer (black), spontaneous ppGpp hydrolysis in buffer in the presence of 4 mM Zn^{2+} (blue), ppGpp in presence of SpoT without (brown) and with Mn^{2+} (purple), respectively. The chromatograms were corrected by subtracting the UV signal of the corresponding buffer injections. **h-m** Purification of SpoT at different pH values and through encapsulation. **h** Purification of 6His-SpoT in Tris buffer, eluted from the Ni-Sepharose column by increasing imidazole concentrations. (LSP: low-speed pellet, SN: supernatant after low-speed centrifugation, FT: flow through, E1-10: elution fraction). **i** A sample of E8 was kept at 4°C overnight showing a ±50% degradation. **j** 6His-SpoT purified in cytosolic buffer remained stable at 4°C for four days. For **i** and **j,** the same size marker was used in the first lane as in **h**. For **h-j** and **l**, 20 µl of the samples were loaded on a 12% SDS gel and stained with Brilliant Blue G250. **k** ppGpp hydrolysis rates (at 25°C in 1 mM $MnCl_2$; slope of a linear regression of the time-dependent ppGpp concentration) by 6His-SpoT decreased over the four days at 4°C. The reaction was started by ppGpp (1 mM) and at different time points samples were taken and mixed with 10 mM EDTA

to stop the reaction (means ±stdevs, n=2). **l** 6His-SpoT was eluted as in **h** with elution buffers adjusted to pH 7.5, pH 8.0, pH 8.5 and 9.0. **m** Encapsulin-SpoT is co-expressed and purified (see Methods). 3 µl were used for SDS PAGE which indicates that SpoT (~80 kDa) has been co-purified with encapsulin (~29 kDa). All gels in **h-j** and **l-m** were run once. Shown are preparations from two independent samples (±3 mg ml^{-1}). Source data are provided as a Source Data file.

Supplementary References

1. Van den Bergh, B. *et al.* Frequency of antibiotic application drives rapid evolutionary adaptation of *Escherichia coli* persistence. *Nat. Microbiol.* **1**, 16020 (2016).

2. Keseler, I. M. *et al.* The EcoCyc database: Reflecting new knowledge about *Escherichia coli* K-12. *Nucleic Acids Res.* **45**, D543–D550 (2017).

3. Zhao, G. & London, E. An amino acid "transmembrane tendency" scale that approaches the theoretical limit to accuracy for prediction of transmembrane helices: Relationship to biological hydrophobicity. *Protein Sci.* **15**, 1987–2001 (2006).

4. Omasits, U., Ahrens, C. H., Müller, S. & Wollscheid, B. Protter: Interactive protein feature visualization and integration with experimental proteomic data. *Bioinformatics* **30**, 884–886 (2014).

5. Chen, S. L. *et al.* Identification of genes subject to positive selection in uropathogenic strains of *Escherichia coli*: a comparative genomics approach. *Proc. Natl. Acad. Sci. U. S. A.* **103**, 5977–5982 (2006).

6. Gnandt, E., Schimpf, J., Harter, C., Hoeser, J. & Friedrich, T. Reduction of the off-pathway iron-sulphur cluster N1a of *Escherichia coli* respiratory complex I restrains NAD+ dissociation. *Sci. Rep.* **7**, 8754 (2017).

7. Datsenko, K. A. & Wanner, B. L. One-step inactivation of chromosomal genes in *Escherichia coli* K-12 using PCR products. *Proc. Natl. Acad. Sci. U. S. A.* **97**, 6640–5 (2000).

8. Bougdour, A. & Gottesman, S. ppGpp regulation of RpoS degradation via anti-adaptor protein IraP. *Proc. Natl. Acad. Sci.* **104**, 12896–12901 (2007).

9. Amarneh, B., De Leon-Rangel, J. & Vik, S. B. Construction of a deletion strain and expression vector for the *Escherichia coli* NADH:ubiquinone oxidoreductase (Complex I). *Biochim. Biophys. Acta* **1757**, 1557–60 (2006).

10. Baba, T. *et al.* Construction of *Escherichia coli* K-12 in-frame, single-gene knockout mutants: the Keio collection. *Mol. Syst. Biol.* **2**, 2006.0008 (2006).

11. Xiao, H. *et al.* Residual guanosine 3',5'-bispyrophosphate synthetic activity of *relA* null mutants can be eliminated by *spoT* null mutations. *J. Biol. Chem.* **266**, 5980–5990 (1991).

12. Schmidt, A. *et al.* The quantitative and condition-dependent *Escherichia coli* proteome. *Nat. Biotechnol.* **34**, 104–110 (2016).

13. Radzikowski, J. L. *et al.* Bacterial persistence is an active σ^S stress response to metabolic flux limitation. *Mol. Syst. Biol.* **12**, 882 (2016).

14. Berg, J., Hung, Y. P. & Yellen, G. A genetically encoded fluorescent reporter of ATP:ADP ratio. *Nat. Methods* **6**, 161–6 (2009).

15. Wilmaerts, D. *et al.* The persistence-inducing toxin HokB forms dynamic pores that cause ATP leakage. *MBio* **9**, 1–12 (2018).

16. Pohl, T., Uhlmann, M., Kaufenstein, M. & Friedrich, T. Lambda Red-mediated mutagenesis and efficient large scale affinity purification of the *Escherichia coli* NADH:ubiquinone oxidoreductase (complex I). *Biochemistry* **46**, 10694–702 (2007).

17. Lončar, N., Rozeboom, H. J., Franken, L. E., Stuart, M. C. A. & Fraaije, M. W. Structure of a robust

bacterial protein cage and its application as a versatile biocatalytic platform through enzyme encapsulation. *Biochem. Biophys. Res. Commun.* **529**, 548–553 (2020).

18. Martinez, K. a *et al.* Cytoplasmic pH response to acid stress in individual cells of *Escherichia coli* and *Bacillus subtilis* observed by fluorescence ratio imaging microscopy. *Appl. Environ. Microbiol.* **78**, 3706–3714 (2012).

19. Baradaran, R., Berrisford, J. M., Minhas, G. S. & Sazanov, L. A. Crystal structure of the entire respiratory complex I. *Nature* **494**, 25–30 (2013).

20. Efremov, R. G. & Sazanov, L. A. Structure of the membrane domain of respiratory complex I. *Nature* **476**, 414–20 (2011).

21. Di Luca, A. *et al.* Symmetry-related proton transfer pathways in respiratory complex I. *Proc. Natl. Acad. Sci.* **114**, E6314–E6321 (2017).
