# Supplementary material for: Mutations in respiratory complex I promote antibiotic persistence through alterations in intracellular acidity and protein synthesis
Source: Nat Commun. 2022 Jan 27;13:546. doi: 10.1038/s41467-022-28141-x (PMC8795404; doi:10.1038/s41467-022-28141-x)
Supplement: Supplementary file 3 — Description of Additional Supplementary Files [file 41467_2022_28141_MOESM3_ESM.pdf]

Description of Additional Supplementary Files

Supplementary Data 1: Mutations >5% in populations of *E. coli*, evolved under recurrent aminoglycoside treatment to high persistence.

[1]Naming is as follows: the first abbreviation denotes the antibiotic that was used during the evolution experiments and the number indicates the population.
[2]Location with regards to the used reference for *E. coli* K12 MG1655 NC_000913.

Supplementary Data 2: Mutations <5% that target the main targets identified before in populations of *E. coli*, evolved under recurrent aminoglycoside treatment to high persistence.
Here, only the target genes identified in Supplementary Data 1 were analyzed for low frequency mutations without any cut-off frequency (thereby potentially containing false positives), supplemented with genes that locate in the same operon if a clear operon structure was available on EcoCyc2. The full list of genes grouped in operons: *nuoABCDCDEFGHIJKLMN, mscL, yaaU, yqhA, kdpDE, astABCDE, oppABCD, gadBC, yhiD, dctA, kch, yobD, accD, ybbW-glxK-ybbY-allB-glxR-hyi-gcl, hycABCDEFGHI, yrhB, mdtABCD-baeSR, mdtEF, crp, manXYZ, mgtA*.

[1]Naming is as follows: the first abbreviation denotes the antibiotic that was used during the evolution experiments and the number indicates the population.
[2]Location with regards to the used reference for *E. coli* K12 MG1655 NC_000913.3.

Supplementary Data 3: Key resources produced and/or used in this study.

Supplementary Data 4: Primers used in this study.
