# Supplementary material for: Mutations in respiratory complex I promote antibiotic persistence through alterations in intracellular acidity and protein synthesis
Source: Nat Commun. 2022 Jan 27;13:546. doi: 10.1038/s41467-022-28141-x (PMC8795404; doi:10.1038/s41467-022-28141-x)
Supplement: Supplementary file 8 — Reporting Summary [file 41467_2022_28141_MOESM8_ESM.pdf]

nature portfolio

Corresponding author(s): Matthias Heinemann; Jan Michiels

Last updated by author(s): Dec 8, 2021

Reporting Summary

Nature Portfolio wishes to improve the reproducibility of the work that we publish. This form provides structure for consistency and transparency in reporting. For further information on Nature Portfolio policies, see our Editorial Policies and the Editorial Policy Checklist.

Statistics

For all statistical analyses, confirm that the following items are present in the figure legend, table legend, main text, or Methods section.

n/a	Confirmed	
☐	☒	The exact sample size (*n*) for each experimental group/condition, given as a discrete number and unit of measurement
☒	☐	A statement on whether measurements were taken from distinct samples or whether the same sample was measured repeatedly
☐	☒	The statistical test(s) used AND whether they are one- or two-sided<br>*Only common tests should be described solely by name; describe more complex techniques in the Methods section.*
☒	☐	A description of all covariates tested
☐	☒	A description of any assumptions or corrections, such as tests of normality and adjustment for multiple comparisons
☐	☒	A full description of the statistical parameters including central tendency (e.g. means) or other basic estimates (e.g. regression coefficient) AND variation (e.g. standard deviation) or associated estimates of uncertainty (e.g. confidence intervals)
☐	☒	For null hypothesis testing, the test statistic (e.g. F, t, r) with confidence intervals, effect sizes, degrees of freedom and P value noted<br>*Give P values as exact values whenever suitable.*
☒	☐	For Bayesian analysis, information on the choice of priors and Markov chain Monte Carlo settings
☒	☐	For hierarchical and complex designs, identification of the appropriate level for tests and full reporting of outcomes
☐	☒	Estimates of effect sizes (e.g. Cohen's *d*, Pearson's *r*), indicating how they were calculated

Our web collection on statistics for biologists contains articles on many of the points above.

Software and code

Policy information about availability of computer code

Data collection	No software was used for data collection
Data analysis	Data analyses software that was used: CLC Genomics Workbench (11.0), FlowJo (10.0.8), Graphpad Prism (8.0), PyMOL (2.0), Visual Molecular Dynamics (VMD, 1.9.3), Structural Alignment of Multiple Proteins (STAMP), NIS-Elements, RStudio (1.4.1717) with R (4.1.0), SWISS-MODEL, Geneious (11.1.5), .Capt software, circos,

For manuscripts utilizing custom algorithms or software that are central to the research but not yet described in published literature, software must be made available to editors and reviewers. We strongly encourage code deposition in a community repository (e.g. GitHub). See the Nature Portfolio guidelines for submitting code & software for further information.

Data

Policy information about availability of data

All manuscripts must include a data availability statement. This statement should provide the following information, where applicable:

- Accession codes, unique identifiers, or web links for publicly available datasets
- A description of any restrictions on data availability
- For clinical datasets or third party data, please ensure that the statement adheres to our policy

The NGS Sequencing data generated in this study have been deposited in the SRA database under the accession codes PRJNA498891 [https://www.ncbi.nlm.nih.gov/bioproject/PRJNA498891], PRJNA498708 [https://www.ncbi.nlm.nih.gov/bioproject/PRJNA498708], PRJNA498717 [https://www.ncbi.nlm.nih.gov/bioproject/PRJNA498717], PRJNA270307 [https://www.ncbi.nlm.nih.gov/bioproject/PRJNA270307], and PRJNA768774 [https://www.ncbi.nlm.nih.gov/bioproject/PRJNA768774]. The Mass spectrometry raw data files have been deposited to the ProteomeXchange Consortium via the PRIDE partner repository under the data set identifier PXD029006 [https://doi.org/10.6019/PXD029006]. The data underlying all the figures generated in this study are

provided in the Source Data file. The Mass spectrometry data used in this study are available via the ProteomeXchange Consortium via the PRIDE partner repository under the data set identifiers PXD000498 [https://doi.org/10.6019/PXD000498] and PXD001968 [https://doi.org/10.6019/PXD001968]. The sequence data used in this study are available in the NCBI nucleotide database (NC_000913.3, NC_007946.1, NC_007941.1), in the Uniprot database (P0AFC3, P0AFC7, P33599, P0AFD1, P31979, P33602, P0AFD4, P0AFD6, P0AFE0, P0AFE4, P33607, P0AFE8, P0AFF0), in the PDB database (6g2j, 4he8) and in the PubChem database (135398637).

Field-specific reporting

Please select the one below that is the best fit for your research. If you are not sure, read the appropriate sections before making your selection.

☒ Life sciences ☐ Behavioural & social sciences ☐ Ecological, evolutionary & environmental sciences

For a reference copy of the document with all sections, see nature.com/documents/nr-reporting-summary-flat.pdf

Life sciences study design

All studies must disclose on these points even when the disclosure is negative.

Sample size	No statistical methods were used to predetermine the sample size. Sample sizes were determined based on the variability of the phenotype and on previous experience in the labs involved.
Data exclusions	Generally, no data was excluded from the analyses unless extreme outliers (based on statistical outlier detection) were detected
Replication	All repeats that are treated in the statistical analyses as biological repeats, were performed during independent runs (other days, other batches of medium etc). For each experiment, each group/timepoint, the replication number can be found in the Source Data file. Some of the experiments that substantiate our main findings were confirmed across different setups, different labs and by different researchers.
Randomization	Samples were generally not randomized as, in most of the assays, all strains/conditions could be measured in the same run Whenever simultaneous assessment of all groups was not possible or (additional) groups were added to the dataset in later runs, we made sure to include a control in all runs of an experiment to enable between-experiment comparisons. Furthermore, we included the information about sample allocation in the statistical analyses (as random factors) were necessary to control for covariates. Randomization within an experimental run (e.g. order of sample manipulation) was not performed as we have no previous indication that this is a major factor of variance in our experiments.
Blinding	Blinding was not applied as we do not expect such biases and because of practical reasons (e.g. sometimes the experimenter needs to know which sample the control is for constructing a calibration curve)

Reporting for specific materials, systems and methods

We require information from authors about some types of materials, experimental systems and methods used in many studies. Here, indicate whether each material, system or method listed is relevant to your study. If you are not sure if a list item applies to your research, read the appropriate section before selecting a response.

Materials & experimental systems

n/a	Involved in the study	
☒	☐	Antibodies
☒	☐	Eukaryotic cell lines
☒	☐	Palaeontology and archaeology
☐	☒	Animals and other organisms
☒	☐	Human research participants
☒	☐	Clinical data
☒	☐	Dual use research of concern

Methods

n/a	Involved in the study	
☒	☐	ChIP-seq
☐	☒	Flow cytometry
☒	☐	MRI-based neuroimaging

Animals and other organisms

Policy information about studies involving animals; ARRIVE guidelines recommended for reporting animal research

Laboratory animals	As organisms, we only used bacteria as described in our table S5
Wild animals	n/a
Field-collected samples	n/a
Ethics oversight	n/a

Note that full information on the approval of the study protocol must also be provided in the manuscript.

Flow Cytometry

Plots

Confirm that:

☒ The axis labels state the marker and fluorochrome used (e.g. CD4-FITC).

☒ The axis scales are clearly visible. Include numbers along axes only for bottom left plot of group (a 'group' is an analysis of identical markers).

☒ All plots are contour plots with outliers or pseudocolor plots.

☒ A numerical value for number of cells or percentage (with statistics) is provided.

Methodology

Sample preparation	Stationary phase bacterial cultures were incubated for 10-20 min with 10 µg ml-1 DiBAC4(3).
Instrument	BD Influx
Software	FlowJo v10.3
Cell population abundance	100 000 were analyzed for each sample
Gating strategy	no gating was applied, histograms in function of dibac fluorescence showed the expected subpopulations with samples and CCCP controls while untreated samples showed no fluorescence at al. Since we show all events, a gating strategy plot was not provided.

☐ Tick this box to confirm that a figure exemplifying the gating strategy is provided in the Supplementary Information.
